# Association of the Stress Hyperglycemia Ratio and Prognosis After Endovascular Treatment: A Systematic Review and Meta‐Analysis

**DOI:** 10.1002/brb3.71245

**Published:** 2026-02-05

**Authors:** Jiayu You, Qianshuo Liu, Xingqiang Li

**Affiliations:** ^1^ Department of Neurology and Neuroscience, Shenyang Brain Institute Shenyang First People's Hospital Shenyang Liaoning China; ^2^ Department of Neurology The Fourth Affiliated Hospital of China Medical University Shenyang Liaoning China

**Keywords:** clinical outcome, endovascular treatment, meta‐analysis, stress hyperglycemia ratio, stroke

## Abstract

**Background and Purpose:**

Stress hyperglycemia (SH) is prevalent in patients with acute ischemic stroke (AIS). The stress hyperglycemia ratio (SHR), calculated as the fasting blood glucose (FBG)/glycosylated hemoglobin (HbA1c) ratio, has been widely used to evaluate SH. However, the correlation between SHR and clinical outcomes in AIS patients with large vessel occlusion (LVO) following endovascular treatment (EVT, including mechanical thrombectomy, contact aspiration, intra‐arterial thrombolysis, excluding intravenous thrombolysis) remains unclear. This study aimed to perform a meta‐analysis to investigate the association between SHR and clinical outcomes in EVT‐treated AIS patients with LVO.

**Methods:**

A comprehensive literature search was conducted across multiple databases, including PubMed, Web of Science, Embase, and the Cochrane Library, to identify studies investigating the association between SHR and clinical outcomes. The entire study was executed in strict adherence to the Preferred Reporting Items for Systematic Reviews and Meta‐Analysis (PRISMA) guidelines. References were screened based on pre‐specified inclusion and exclusion criteria, and the Newcastle–Ottawa Scale (NOS) was rigorously applied to assess potential bias risks in the selected studies. RevMan 5.3 software was utilized for performing meta‐analyses on the included literature.

**Results:**

A total of 10 studies met the inclusion criteria, and 3646 AIS patients who underwent EVT were included in this analysis. The meta‐analysis results demonstrated a higher SHR was associated with an increased risk of poor outcomes (modified Rankin Scale [mRS] 3–6) at 90 days (odds ratio [OR] = 2.65, 95% CI: 2.30–3.06, *p* < 0.001), mortality (OR = 2.62, 95% CI: 1.81–3.79, *p* < 0.001), intracranial hemorrhage (ICH) (OR = 1.53, 95% confidence interval [CI]: 1.27–1.85, *p* < 0.001), symptomatic intracranial hemorrhage (sICH) (OR = 2.05, 95% CI: 1.27–3.30, *p* < 0.003) after EVT.

**Conclusion:**

A higher SHR may increase the occurrence of poor outcomes, mortality, ICH, and sICH in AIS patients caused by LVO after EVT. SHR is associated with poor prognosis in AIS patients caused by LVO after EVT.

## Graphical abstract

A comprehensive electronic literature search was performed in PubMed, Web of Science, Embase, and the Cochrane Library following the PRISMA guidelines. After screening by pre‐defined inclusion/exclusion criteria and methodological quality assessment using the Newcastle–Ottawa Scale (NOS), 10 eligible studies involving 3646 LVO‐AIS patients who received EVT were included. Meta‐analysis via RevMan 5.3 software revealed that elevated SHR was significantly associated with increased risks of 90‐day poor functional outcomes (modified Rankin Scale [mRS] 3‐6, odds ratio [OR] = 2.65, 95% confidence interval [CI]:2.30‐3.06, *p* < 0.001), all‐cause mortality (OR = 2.62, 95% CI: 1.81‐3.79, *p* < 0.001), intracranial hemorrhage (ICH, OR = 1.53, 95% CI: 1.27‐1.85, *p* < 0.001), and symptomatic intracranial hemorrhage (sICH, OR = 2.05, 95% CI: 1.27‐3.30, *p* = 0.003). Collectively, SHR can serve as a reliable prognostic indicator for LVO‐AIS patients after EVT, providing valuable reference for clinical risk stratification and management.

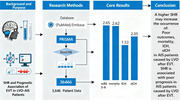

## 1 Introduction

Acute ischemic stroke (AIS) ranks as the second leading cause of death worldwide and a primary contributor to long‐term disability (Kim et al. 2019). Substantial evidence has established that endovascular treatment (EVT) effectively reduces 90‐day disability and improves clinical outcomes in AIS patients with large vessel occlusion (LVO). EVT is thus widely recommended as the preferred treatment for such patients (Goyal et al. 2016; Goyal et al. 2015; Saver et al. 2015).

Stress hyperglycemia (SH), defined as transient hyperglycemia secondary to neurohormonal dysregulation and inflammatory response, may impact clinical outcomes in AIS patients (Capes et al. 2001). AIS patients often exhibit SH at hospital admission, which can be attributed to suboptimal chronic hyperglycemia control, physiological stress, or a combination of both. However, preexisting hyperglycemia complicates the identification of SH in diabetic patients. Glycosylated hemoglobin (HbA1c) reflects average baseline glucose levels over the previous 2–3 months (Welsh et al. 2020). Multiple studies have associated HbA1c with poor outcomes in EVT‐treated AIS patients (Diprose et al. 2020; Chang et al. 2021), though some research suggests HbA1c may not reliably predict unfavorable neurological outcomes (Sung et al. 2017).

The stress hyperglycemia ratio (SHR), defined as the fasting blood glucose (FBG) to HbA1c ratio, was first introduced by Roberts et al. (2015) for assessing SH. Studies have shown that SHR is a more robust predictor of critical illness than FBG or HbA1c alone. Notably, SHR has been associated with futile recanalization in patients who achieved successful revascularization after EVT (Merlino et al. 2024). Additionally, research has linked SHR to an increased risk of recurrent stroke in patients with minor ischemic stroke or transient ischemic attack (Pan et al. 2017), while a high SHR has been identified as an indicator of poor prognosis in AIS patients (Huang et al. 2022).

Despite growing evidence on the role of the SHR in stroke outcomes, no meta‐analysis has yet comprehensively evaluated its association with clinical outcomes in AIS patients with LVO who undergo EVT. To address this gap, we conducted a systematic review and meta‐analysis. Given that admission FBG and HbA1c levels are readily available in acute care settings across numerous countries, SHR has the potential to become a practical and widely applicable tool for predicting clinical prognosis in routine clinical practice.

### 1.1 Methods and PMO Statement

This systematic review was conducted in accordance with the Preferred Reporting Items for Systematic Reviews and Meta‐Analyses (PRISMA) statement and Cochrane guidelines for systematic reviews of interventions. The PMO (Population, Measurement, Outcomes) framework was defined as follows: (1) Population: AIS patients with LVO treated with EVT. (2) Measurement: SHR was calculated as the admission glucose‐to‐HbA1c ratio. Patients were categorized into relatively low versus high SHR groups based on study‐specific stratification: for example, in studies with three groups, the first group was defined as low SHR, and the latter two as high SHR; in studies with four groups, the first two groups were classified as low SHR, and the remaining two as high SHR. (3) Outcomes: Poor functional outcome was defined as a modified Rankin Scale (mRS) score ≥3 at 90 days. Mortality during follow‐up and symptomatic intracerebral hemorrhage (sICH) were also evaluated. sICH was defined as intracerebral hemorrhage accompanied by a ≥4‐point deterioration in the National Institutes of Health Stroke Scale (NIHSS) score (Hacke et al. 1998), while ICH was defined as any post‐EVT hemorrhagic transformation.

### 1.2 Literature Search Strategy

A systematic search for English‐language articles was carried out. Two reviewers (Liu QS, Li XQ) systematically screened the electronic databases from PubMed, Embase, and the Cochrane Library up to March 2025. The following search strategy was used across these databases: ((“stress hyperglycemia”[all fields]) OR (“stress hyperglycemia ratio”[all fields]) OR (“hyperglycemia”[all fields]) OR (“stress”[all fields]) OR (“diabetes”[all fields]) OR (“glycosylated hemoglobin”[all fields]) OR (“glycosylated hemoglobin”[all fields])) AND ((“stroke”[all fields]) OR (“ischemic stroke”[all fields])) AND ((“endovascular treatment”[all fields]) OR (“mechanical thrombectomy”[all fields]) OR (“thrombectomy”[all fields]) OR (“inter artery therapy”[all fields]) OR (“solitaire”[all fields]) OR (“stent‐retriever”[all fields]) OR (“reperfusion therapy”[all fields])).

### 1.3 Inclusion and Exclusion Criteria

All potential studies were independently screened and evaluated for inclusion/exclusion criteria by two reviewers (You JY, Li XQ). Study selection adhered to the following strict criteria: (1) Publication Criteria: Only articles published in peer‐reviewed journals with established impact were included, ensuring scientific rigor through adherence to formal editorial and review processes. (2) Participant Characteristics: Studies were included if they enrolled AIS patients with acute LVO confirmed by computed tomography angiography (CTA), magnetic resonance angiography (MRA), or digital subtraction angiography (DSA), with symptom onset to groin puncture time ≤24 h and no intracranial hemorrhage on initial CT. Patients received or were scheduled to receive EVT (including mechanical thrombectomy, contact aspiration, intra‐arterial thrombolysis, etc.) or EVT combined with intravenous thrombolysis (IVT). Studies were required to report FBG and HbA1c data, with venous samples collected within 24 h of admission to capture acute metabolic status. SHR was calculated as FBG (mmol/L)/HbA1c (%). (3) Comparison Strategy: Analyses compared relatively low versus high SHR groups, with stratification defined per study (e.g., first group as low SHR in three‐group studies, first two groups as low SHR in four‐group studies). (4) Outcome Measures: Primary outcomes included poor functional outcome (mRS ≥3 at 90 days), mortality, sICH, and any post‐EVT ICH.

To ensure data homogeneity and quality, the following were excluded from the analysis: case reports, review articles, notes, meta‐analyses, editorials, letters to the editor, commentaries, conference abstracts, and non‐English studies.

### 1.4 Data Extraction

Two reviewers (Liu QS, You JY) independently extracted data using standardized forms. The following information was abstracted from included studies: (1) Basic Study Characteristics: study identifier (publication year + first author), country, study design, sample size; (2) Participant Characteristics: male proportion, stroke subtype, EVT modality, primary/secondary endpoints, follow‐up duration; (3) Outcome Data: relevant outcomes of interest, etc.

### 1.5 Risk of Bias Assessment

The Newcastle–Ottawa Scale (NOS) (Stang 2010) was used to assess potential bias risks in included studies. The NOS evaluation framework comprised three domains (each with a maximum of 3 points): (1) Study Selection: evaluating sample representativeness and adequacy of inclusion/exclusion criteria; (2) Study Comparability: assessing control for confounding variables across study groups; (3) Outcome Assessment: evaluating the validity and reliability of exposure and outcome measurements. Studies were scored on a 9‐point scale, with scores >6 indicating high methodological quality and low bias risk. Three reviewers (Li XQ, You JY, Liu QS) independently performed the assessment, and discrepancies were resolved through group discussion.

### 1.6 Statistical Analysis

For comparisons of outcomes between high‐ and low‐SHR groups, we calculated odds ratios (ORs) with 95% confidence intervals (CIs). Given potential clinical heterogeneity across studies, meta‐analyses were performed using the DerSimonian–Laird random‐effects model. A two‐sided *p* < 0.05 was considered statistically significant. Heterogeneity was evaluated via the Cochrane Q test, with *p* < 0.1 or *I* ^2^ > 50% indicating substantial heterogeneity. In accordance with a priori defined SHR cutoffs, group‐specific data (high/low SHR) were extracted from each study. Publication bias was assessed using funnel plot analysis. All statistical analyses were conducted with Review Manager (RevMan) 5.3 software.

## 2 Results

**FIGURE 1:**
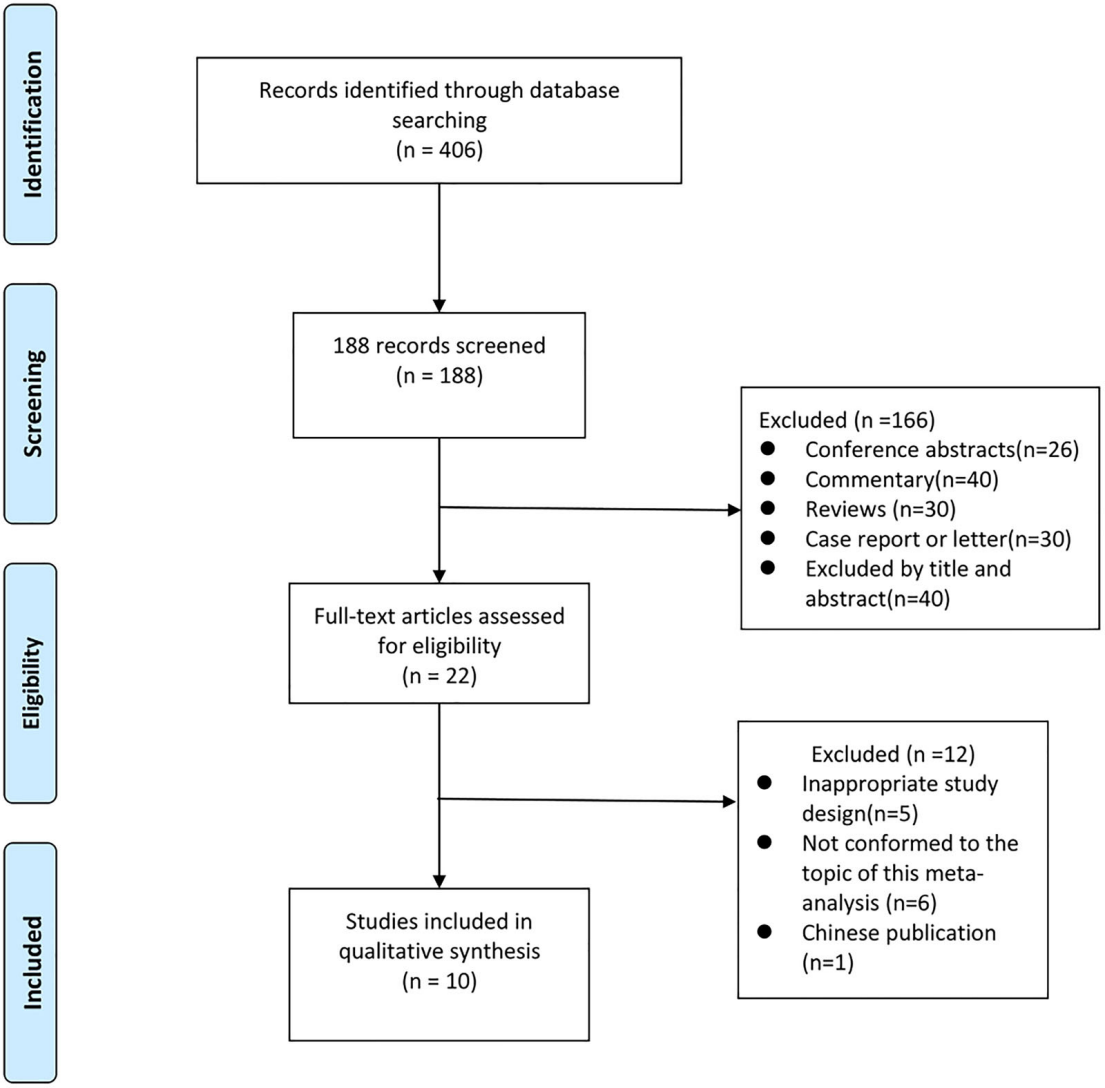
PRISMA flow‐chart for selection of studies included in the meta‐analysis.

A literature search was conducted across PubMed, Embase, and Cochrane Library databases. Initially, 406 records were identified, followed by full‐text evaluation of 22 articles. Twelve studies were excluded: 5 for inappropriate design (non‐randomized or cross‐sectional designs not meeting study criteria), 6 for irrelevant topics (not directly addressing the SHR‐stroke outcome association), and 1 due to Chinese publication (as inclusion was limited to English‐language studies). Ultimately, 10 studies (Wang et al. 2019; Chen et al. 2019; Merlino et al. 2021; Duan et al. 2023; Peng et al. 2023; Wang and Fan 2023; Zhang et al. 2023; Peng et al. 2024; Yang et al. 2024; Sun et al. 2023) were included, comprising 8 single‐center retrospective and 2 multicenter studies with 3,646 total patients. A PRISMA‐compliant flowchart is shown in Figure 1, and key study characteristics are summarized in Table 1.

**TABLE 1:** Baseline characteristic description of the included studies.

Reference	Country	Study design	Participants	Males (%)	Operation	Primary endpoint	Second endpoint	Clinical follow‐up
Wang et al. (2019)	China	Retrospectively multicenter	321	61.1	Mechanical thrombectomy	Poor outcome	—	3 months
Chen et al. (2019)	China	Retrospectively single‐center	160	67.5	Mechanical thrombectomy	Poor outcome	—	3 months
Merlino et al. (2021)	Italy	Retrospectively single‐center	204	49.0	Mechanical thrombectomy	Poor outcome	Mortality	3 months
Duan et al. (2023)	China	Retrospectively single‐center	576	56.9	Endovascular treatment	Poor outcome	—	3 months
Peng et al. (2023)	China	Multicenter, double‐blind RCT	542	56.5	Endovascular treatment	Favorable outcome	Excellent outcome	3 months
Wang and Fan (2023)	China	Retrospectively single‐center	209	54.5	Endovascular treatment	Mortality	Poor outcome	3 months
Zhang et al. (2023)	China	Retrospectively single‐center	408	65.0	Mechanical thrombectomy	Excellent outcome	Mortality	3 months
Peng et al. (2024)	China	Prospective single‐center	250	75.2	Endovascular treatment	Favorable outcome	Mortality	3 months
Yang et al. (2024)	China	Retrospectively single‐center	553	66.5	Mechanical thrombectomy	Mortality	Poor outcome	3 months
Sun et al. (2023)	China	Retrospectively single‐center	423	59.1	Mechanical thrombectomy	Poor outcome	—	3 months

### 2.1 Heterogeneity

Based on the study results, moderate to substantial statistical heterogeneity was detected across several outcomes. For poor functional outcome at 90 days, the Cochran Q test showed a *p*‐value of 0.05 and an *I* ^2^ of 48%; for mortality, the Cochran *Q* test yielded a *p*‐value of 0.0003 with an *I* ^2^ of 71%; for ICH, the *p*‐value was 0.44 and *I* ^2^ = 0%; for sICH, the *p*‐value was 0.05 and *I* ^2^ = 54%. In response to these findings, a random‐effects model was applied for mortality and sICH, whereas a fixed‐effects model was used for poor outcome and ICH in subsequent meta‐analyses.

### 2.2 Meta‐Analysis of Different Outcomes

**FIGURE 2:**
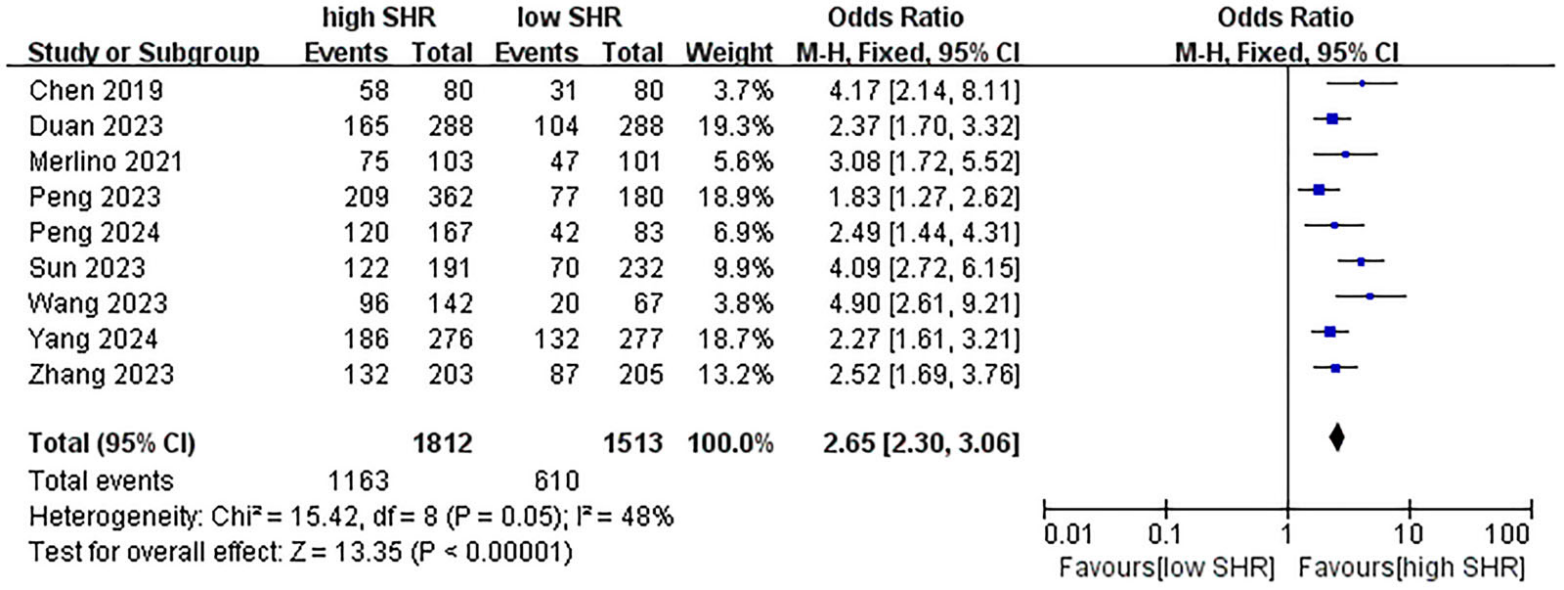
Forest plot showing the association between SHR and poor outcome (mRS ≥ 3 points) at 3 months. M–H, Mantel–Haenszel; CI, confidence interval.

**FIGURE 3:**
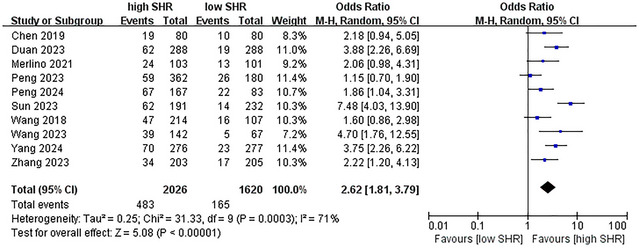
Forest plot showing the association between SHR and mortality.

**FIGURE 4:**
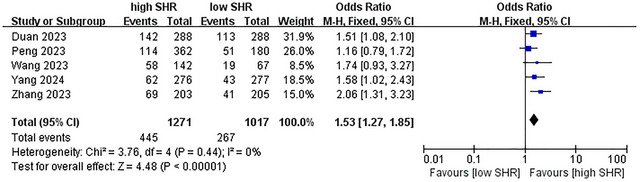
Forest plot showing the association between SHR and ICH.

**FIGURE 5:**
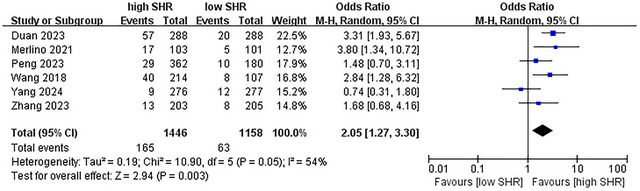
Forest plot showing the association between SHR and sICH.

The results are summarized in Table 2. The meta‐analysis revealed a significant increase in the incidence of several outcomes. For poor outcome at 90 days, OR = 2.65 (95% CI: 2.30–3.06, *p* < 0.001, *I* ^2^ = 48%; 9 studies were included, see Figure 2) (Chen et al. 2019; Merlino et al. 2021; Duan et al. 2023; Peng et al. 2023; Wang and Fan 2023; Zhang et al. 2023; Peng et al. 2024; Yang et al. 2024; Sun et al. 2023); for mortality, OR = 2.62 (95% CI: 1.81–3.79, *p* < 0.001, *I* ^2^ = 71%; 10 studies were included, see Figure 3) (Wang et al. 2019; Chen et al. 2019; Merlino et al. 2021; Duan et al. 2023; Peng et al. 2023; Wang and Fan 2023; Zhang et al. 2023; Peng et al. 2024; Yang et al. 2024; Sun et al. 2023); for ICH, OR = 1.53 (95% CI: 1.27–1.85, *p* < 0.001, *I* ^2^ = 0%; 5 studies were included, see Figure 4) (Duan et al. 2023; Peng et al. 2023; Wang and Fan 2023; Zhang et al. 2023; Yang et al. 2024); for sICH, OR = 2.05 (95% CI: 1.27–3.30, *p* < 0.003, *I* ^2^ = 54%; 6 studies were included, see Figure 5) (Wang et al. 2019; Merlino et al. 2021; Duan et al. 2023; Peng et al. 2023; Zhang et al. 2023; Yang et al. 2024). These results suggest a strong association between high SHR and adverse outcomes.

**TABLE 2:** Heterogeneity and meta‐analysis of included studies.

Items	Trials, *n*	OR	*p*‐value	Heterogeneity (*I* ^2^, *p* for Cochran Q)
Poor outcome	9	2.65	*p* < 0.00001	*I* ^2^ = 48%, *p* = 0.05
Mortality	10	2.62	*p* < 0.00001	*I* ^2^ = 71%, *p* = 0.0003
ICH	5	1.53	*p* < 0.00001	*I* ^2^ = 0%, *p* = 0.44
sICH	6	2.05	*p* = 0.0003	*I* ^2^ = 54%, *p* = 0.05

### 2.3 Sensitivity Analysis

We conducted a sensitivity analysis to test the stability and sensitivity of the included studies by altering the effects model. After changing the effect model, the majority of the subgroup studies remained statistically significant, and the overall conclusions did not change. This indicated that the results of this meta‐analysis were stable. When the random‐effects model was changed to a fixed‐effect model, the analysis showed that a higher SHR was associated with a higher mortality (OR = 2.64, 95% CI: 2.18–3.19, *p* < 0.001), a higher rate of sICH (OR = 2.21, 95% CI: 1.63–3.00, *p* < 0.001). Conversely, when the fixed‐effect model was changed to a random‐effects model, the results demonstrated that a higher SHR was associated with a higher rate of poor prognosis (mRS 3–6) at 90 days (OR = 2.76, 95% CI: 2.25–3.40, *p* < 0.001) and a higher risk of ICH after EVT (OR = 1.53, 95% CI: 1.27–1.85, *p* < 0.001).

### 2.4 Risk of Bias Assessment

**FIGURE 6:**
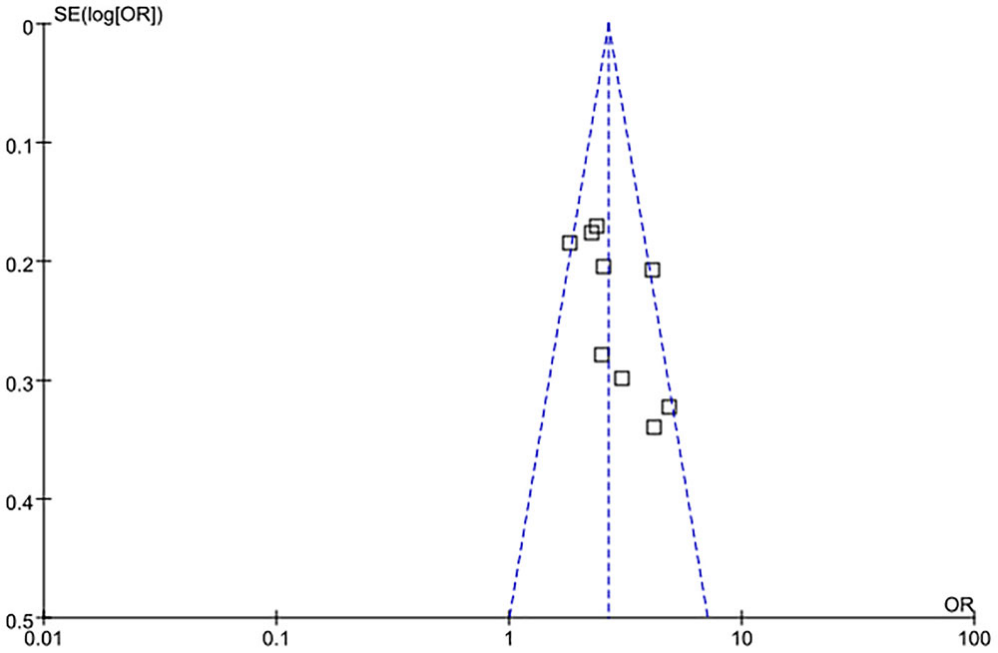
Funnel plots of publication bias.

Funnel plots were employed to detect potential publication bias. The results of the funnel plot analysis demonstrated a relatively symmetrical distribution of the included studies (Figure 6). Furthermore, a quantitative analysis of publication bias using Egger's test indicated that the nine articles included in this study were free from obvious publication bias (*p* > 0.1).

### 2.5 The Quality Assessment of Included Studies

Since all the studies were retrospective in nature, they were evaluated by NOS tool. On average, the studies received a score of 8.3 stars with a standard deviation (SD) of 0.67 stars. The methodological quality of each included study, as assessed by the NOS tool, is presented in Table 3, providing a detailed overview of the potential biases in each study and allowing readers to judge the reliability of the results.

**TABLE 3:** Quality assessment of included studies by NOS.

Study ID	Year	Selection				Comparability		Exposure			Stars
		Q1	Q2	Q3	Q4	Q5	Q6	Q7	Q8	Q9	
Wang et al.	2018	*	*	*	*	*	*	*	*	*	9
Chen et al.	2019	*	*	*	*	*	*	*	*	*	9
Merlino et al.	2021	*	*	*	*	*	*	*	*	*	9
Duan et al.	2023	*	*	*	*	*	*	*	*	*	9
Peng et al.	2023	*	*	—	*	*	*	*	*	*	8
Wang et al.	2023	*	*	—	*	*	*	*	*	*	8
Zhang et al.	2023	*	*	—	*	*	*	*	—	*	7
Sun et al.	2023	*	*	*	*	*	*	*	*	—	8
Peng et al.	2024	*	*	—	*	*	*	*	*	*	8
Yang et al.	2024	*	*	—	*	*	*	*	*	*	8

## 3 Discussion

It is well‐documented that admission hyperglycemia is associated with adverse outcomes in AIS patients with LVO following EVT (Kim et al. 2016; Osei et al. 2017; Goyal et al. 2018; Chamorro et al. 2019). However, admission FBG levels reflect only a transient glucose status, rendering them insufficient to fully characterize disease‐induced metabolic disruption. Regardless of pre‐existing diabetes, the deviation of glucose levels from individual baselines may be more clinically relevant. Multiple studies have shown that, in EVT‐treated AIS patients with LVO, illness‐induced dynamic glucose changes serve as a more robust predictor of poor outcomes than baseline hyperglycemia alone (Yong and Kaste 2008; Merlino et al. 2020). Notably, a substantial proportion of AIS patients experience a stress response, manifesting as acute elevation of plasma glucose levels above baseline—a phenomenon termed stress hyperglycemia (SH).

SH arises from activation of the hypothalamic‐pituitary axis and sympatho‐adrenal system, coupled with the influence of pro‐inflammatory cytokines. These mechanisms drive excessive gluconeogenesis, glycogenolysis, insulin resistance, endothelial apoptosis, and oxidative stress (Dungan et al. 2009). The magnitude of SH varies with patient‐specific factors (e.g., baseline glucose tolerance), disease characteristics (e.g., stroke subtype and severity), and illness stage. A sudden stroke onset disrupts cytokine production and hormonal homeostasis, leading to hepatic glucose overproduction and insulin resistance. SH development involves complex interactions among counter‐regulatory hormones (e.g., catecholamines, growth hormone) and pro‐inflammatory cytokines (Marik and Bellomo 2013). This primarily manifests as hepatic glucose overproduction via gluconeogenesis and upregulated hormones that suppress peripheral glucose uptake. Notably, SH is mediated by profound inflammatory and neuroendocrine dysregulation, distinct from the chronic hyperglycemia of diabetes. Moreover, hyperglycemia exacerbates cytokine release, inflammation, and oxidative stress, creating a vicious cycle that further elevates glucose levels.

The stress hyperglycemia ratio (SHR), calculated as FBG/HbA1c, serves to exclude undiagnosed diabetes, identify stress‐related glycemic exacerbation in diabetic patients, and quantify SH more effectively (Nathan et al. 2007). Recent meta‐analyses have explored the association between SHR and outcomes in AIS patients. In 2022, Huang et al. demonstrated via meta‐analysis that higher SHR was significantly associated with increased odds of poor clinical outcomes, mortality, and neurological deficits (Huang et al. 2022). A 2023 meta‐analysis by the same group further revealed that cumulative high SHR exhibits a non‐linear dose‐response relationship with adverse outcomes and mortality in AIS patients (Huang et al. 2023). In 2024, Jiang et al. (2024) reported that acute‐phase high SHR is an independent predictor of poor prognosis, including functional disability and mortality. Notably, no systematic review and meta‐analysis have yet addressed the association between SHR and clinical outcomes in AIS patients with LVO treated with EVT. To fill this evidence gap, we conducted this meta‐analysis including 3,646 patients. Sensitivity analyses confirmed result stability, though it should be emphasized that most included studies were retrospective, potentially introducing selection and information biases.

Our meta‐analysis demonstrated that higher SHR is associated with poor functional outcomes at 90 days post‐EVT. This finding aligns with prior studies showing that elevated SHR is associated with adverse 3‐month outcomes after mechanical thrombectomy (Chen et al. 2019; Merlino et al. 2021). However, Wang and Fan (2023) reported that severe SH independently elevates the odds of futile recanalization and 3‐month all‐cause mortality in non‐diabetic AIS patients undergoing EVT. The discrepancy might be attributed to the fact that 80% of diabetic patients received pre‐stroke standard glycemic therapy, potentially conferring greater tolerance to stress‐induced glycemic fluctuations compared to non‐diabetic individuals (Chang et al. 2018). Notably, a 2023 study by Duan et al. (2023) revealed that high SHR was more strongly associated with poor prognosis in non‐diabetic stroke patients than in those with pre‐existing diabetes. This may reflect long‐term vascular adaptations to chronic hyperglycemia in diabetic patients, which could mitigate the acute neurovascular injury induced by stress‐related glucose surges.

Our meta‐analysis further confirmed that elevated SHR is associated with increased mortality following EVT. This finding is consistent with Wang et al.’s (2019) research on mortality risk, which reported that higher SHR was associated with increased mortality after mechanical thrombectomy in AIS patients (odds ratio [OR] = 3.01, 95% confidence interval [CI]: 1.06–8.53). Additionally, Zhu et al. (2019) demonstrated in non‐diabetic stroke patients that SHR was linked to heightened risks of stroke recurrence and all‐cause mortality. Concurrently, numerous studies have observed an association between elevated SHR and mortality in AIS populations (Merlino et al. 2021; Li et al. 2020; Merlino et al. 2021).

Our meta‐analysis further indicated that elevated SHR may increase the risk of ICH and sICH following EVT. This aligns with Tian et al.’s (2022) finding that SHR predicts hemorrhage transformation after EVT. Two 2022 studies in hemorrhagic stroke populations showed SHR was a reliable predictor of early hematoma expansion and independently associated with poorer functional outcomes at 3‐month discharge in ICH patients (Chu et al. 2022; Li et al. 2022). Additionally, a 2025 study by Currò et al. (2025) demonstrated SHR may predict early neurological deterioration in spontaneous ICH cases.

The SHR is a simple and readily accessible biomarker for predicting clinical outcomes. Owing to its convenience and non‐invasiveness, SHR holds promise for widespread clinical use in evaluating outcomes and adverse events in AIS patients. Future studies may explore SHR as a component of prediction models or artificial intelligence algorithms for forecasting outcomes in AIS. As an independent prognostic factor, SHR enables clinicians to identify stroke patients at high risk of poor outcomes. In summary, this meta‐analysis comprehensively synthesized existing evidence comparing SHR levels in AIS patients with LVO treated with EVT. We found that higher SHR is associated with increased risks of poor functional outcome, mortality, sICH, and ICH following EVT in LVO patients.

### 3.1 Limitations

Our meta‐analysis has several significant limitations. First, most of the included studies are retrospective studies which may vary in methodological rigor. Even with NOS scoring to assess quality, a score > 6 does not fully eliminate differences in study conduct. Second, we only included English‐language studies, which may exclude potential evidence from non‐English publications and thus introduce a certain degree of language bias. Moreover, most of the included studies were from China, which may affect the generalizability of our findings. Third, the possible mechanisms underlying the association between SH and clinical outcomes, especially the role of early glucose level control, have not been comprehensively explored in this meta‐analysis. Fourth, patients were categorized into relatively low versus high SHR groups based on study‐specific stratification and not all included studies explicitly reported the minimum fasting duration (including abstinence from intravenous glucose administration) for fasting blood glucose measurement. This introduces heterogeneity and limits comparability. Finally, the Egger's test used to assess publication bias may lack sufficient statistical power, given only 9–10 studies were included. Thus, the results of this publication bias test should be interpreted with caution, and our assessment of publication bias remains preliminary.

## 4 Conclusion

A higher SHR significantly increased the occurrence of poor outcomes, mortality, ICH, and sICH in AIS patients caused by LVO after EVT. SHR may be associated with poor prognosis in AIS patients caused by LVO after EVT.

## Author Contributions

Xingqiang Li and Jiayu You conceptualized the study; Qianshuo Liu and Jiayu You developed the methodology; Qianshuo Liu conducted formal analysis; Jiayu You and Qianshuo Liu curated the data; Jiayu You and Qianshuo Liu drafted the original manuscript; all authors revised and edited the manuscript; Xingqiang Li supervised the study and provided resources; all authors validated the study content, approved the final manuscript, and agreed to be accountable for all aspects of the work.

## Conflicts of Interest

The authors have no competing interests.

## Funding

The authors have nothing to report.

## Data Availability

The data supporting the findings of this meta‐analysis are derived from previously published studies. All relevant data are publicly available within the original articles cited in the References section of this manuscript. Individual participant data (IPD) were not accessed or generated in this study, as the analysis was based on aggregated results extracted from eligible literature. Correspondence regarding data from the included primary studies should be directed to the respective authors of those original publications.
